# Short-term outcomes of the right ventricle to pulmonary artery conduit “NeoCor” in Syria: A case series

**DOI:** 10.1016/j.ijscr.2025.111980

**Published:** 2025-09-23

**Authors:** Rahaf Massoud, Sarah Massoud, Yaman Al-Mkaky, Yaman Wazir, Yahya Ibrahim, Alwaleed Al-Dairy

**Affiliations:** a Faculty of Medicine, Damascus University, Syria; b Cardiac Surgery at Faculty of Medicine, Damascus University, Syria

**Keywords:** Congenital heart defects, RV–PA continuity, Bovine pericardial xenograft conduit, RVOT reconstruction, Pediatric cardiac surgery, Case series

## Abstract

**Background:**

Right ventricle–to–pulmonary artery (RV–PA) reconstruction often requires a valved conduit; however, no single option is ideal across ages and anatomical variations. We report early outcomes of a bovine pericardial xenograft conduit (NeoCor) as a practical solution in a resource-limited setting.

**Methods:**

In this single-center retrospective series, eight pediatric patients (1–14 years) underwent RV–PA reconstruction (2019–2024) using a bovine pericardial xenograft conduit sized by nomogram (10–21 mm). A single surgical team performed all procedures under standardized perioperative protocols with intraoperative echocardiography. Complications were graded using standardized criteria. The primary outcome was clean follow-up (no mortality, no reintervention on the conduit/RVOT, no ≥moderate conduit regurgitation, peak Doppler gradient ≤30 mmHg, and no echogenic calcification).

**Results:**

Indications included TGA/VSD/PS (*n* = 4), tetralogy of Fallot with absent pulmonary valve (*n* = 2), VSD–PA with confluent PAs/PDA (*n* = 1), and truncus arteriosus redo (n = 1). Follow-up ranged 6 months–5 years (mean 2 years). All patients met the clean follow-up definition. Conduit Doppler gradients were 5–8 mmHg perioperatively and 8–12 mmHg at follow-up; no calcification or ≥ moderate regurgitation was observed.

**Conclusions:**

A bovine pericardial xenograft conduit is a feasible, accessible option for RV–PA reconstruction with promising short-term hemodynamics in this context. Larger prospective studies and/or registry participation are warranted to evaluate durability, reintervention rates, and cost-effectiveness.

## Highlights

• NeoCor as a cost-effective alternative
• Responding to resource constraints
• Surgical efficiency and customization

## 1 Introduction

Congenital heart defects (CHDs) remain a leading cause of mortality in infants and children [1]**.** Among these, right ventricle to pulmonary artery (RV-PA) discontinuity represents a rare and complex configuration, often associated with severe forms of CHD such as tetralogy of Fallot or pulmonary atresia [2]. The absence of a direct connection between the right ventricle and the pulmonary artery leads to significant hemodynamic challenges, necessitating surgical intervention to restore effective pulmonary blood flow [3]**.** Over the past several decades, advances in surgical techniques have significantly reduced mortality rates. One of these interventions is the use of an RV-PA conduit, which creates an artificial connection to bypass the anatomical discontinuity. However, the selection of the most appropriate conduit remains a major challenge for surgeons, as no ideal graft currently exists. The choice must be tailored to each patient's specific anatomy, age, and clinical needs to maximize outcomes [4]**.** Homografts, particularly aortic (AHG) and pulmonary (PHG) homografts, have been considered the gold standard for right ventricular outflow tract (RVOT) reconstruction for approximately 50 years. Their favorable handling properties, such as ease of suturing and compressibility, make them reliable options [5]**.** Nevertheless, homografts are limited by their tendency to calcify, develop high gradients, and become insufficient over time [6]. Furthermore, their availability in small sizes (less than 16 mm) is often restricted, posing additional challenges in neonates and infants [7]**.** Xenografts offer a viable alternative, encompassing both stentless (bovine jugular vein (Contegra), porcine aortic root (Freestyle)) and stented (Hancock valve) options. The main advantages of xenografts are abundant supply, availability in smaller sizes suitable for neonates, lower cost compared to homografts, and excellent handling characteristics. However, certain types, such as the Hancock conduit (the first heterograft to develop), may be less suitable for the smallest patients because of the stiffness of the Dacron housing, which makes it technically difficult to suture to the pulmonary arteries and right ventricle [8]**.** Despite these advances, RV-PA conduits are still associated with significant restrictions, loss of valve function, conduit narrowing over time, and lack of growth capacity [9]**.** Additionally, the cost and availability of both homografts and xenografts vary widely between countries [10]**.** Most patients will require conduit replacement within 4 to 5 years, mainly due to size mismatch as the child grows. Each subsequent surgery increases the risk of complications, particularly with repeated sternotomies and the presence of an RV-PA conduit [11]**.** In this context, we report our experience with a bovine pericardial xenograft conduit **(NeoCor)** used for RVOT reconstruction in a resource-limited setting, aiming to share early outcomes and technical considerations of this approach.

## 2 Methods

### 2.1 Study design and patients

This is a retrospective descriptive single-center case series. It includes all pediatric patients (ages 1–14 years) who underwent RV–PA reconstruction using a bovine pericardial xenograft conduit (NeoCor; CJSC NeoCor, Kemerovo, Russia) **[**Table 1**]**. From 2019 to 2024, eight consecutive patients underwent open-heart surgery with this approach. Patient weights ranged from 7.5 to 40 kg. Conduit diameter was determined using a standard nomogram (10–21 mm) **[**Table 2**]**. Demographic, clinical, surgical, and follow-up data were collected retrospectively from medical records. Patient confidentiality and anonymity were maintained throughout; all data were securely stored and accessible only to the research team. The work has been reported in line with the SCARE criteria [12] and the PROCESS criteria [13].

**Table 1:** demographics of the sample.

Variable	Category	Number of patients
Age	1 year	3 patients
	2 years	1 patient
	4 years	1 patient
	5 years	2 patients
	14 years	1 patient
Sex	Males	50 %
	Females	50 %
Weight	<10 kg	3 patients
	10–20 kg	4 patients
	>20 kg	1 patient

**Table 2:** characteristics of the conduits.

Diameter of the conduit	Number of Patients
10 mm	1
12 mm	3
14 mm	2
16 mm	1
21 mm	1

### 2.2 Device description

In this study, “NeoCor conduit” denotes a pre-manufactured bovine pericardial xenograft conduit (not a hand-sewn conduit fabricated intraoperatively).

### 2.3 Surgical strategies

**Fig. 1:**
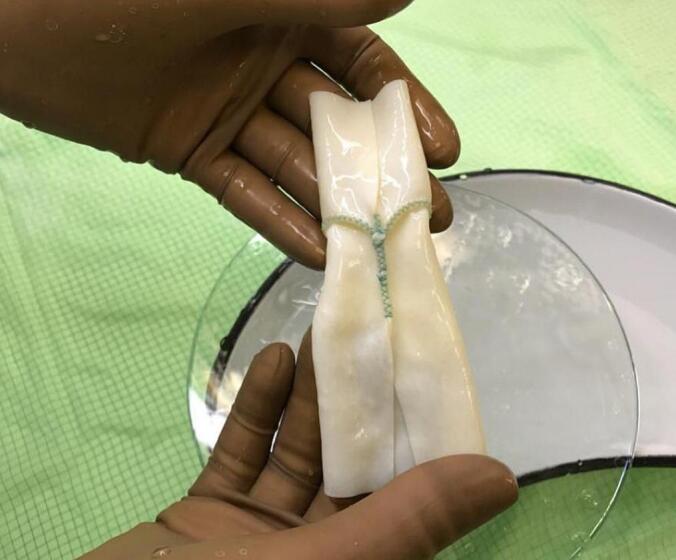
Bovine pericardial xenograft valved conduit prepared for implantation, illustrating the tubular structure and integrated valve leaflets prior to anastomosis between the RV outflow tract and the pulmonary artery.

**Fig. 2:**
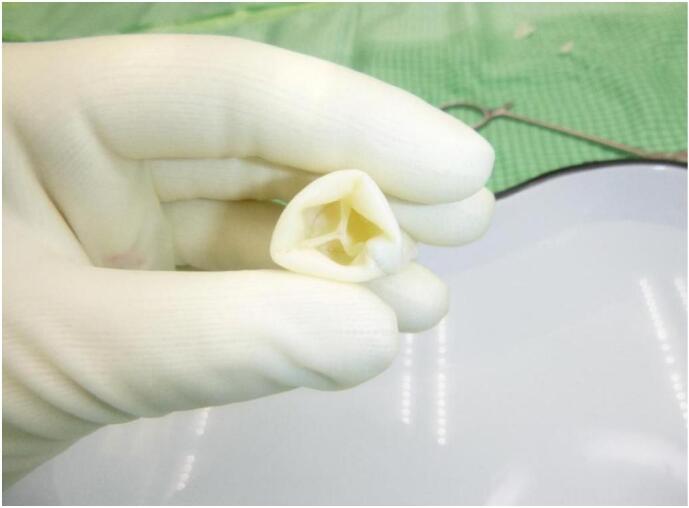
Intraoperative photograph of the bovine pericardial xenograft valved conduit (NeoCor) showing the trileaflet valve morphology within the conduit lumen.

All procedures were performed by the same team of experienced congenital cardiac surgeons. Operating room preparations followed standardized institutional protocols. After establishing cardiopulmonary bypass, the bovine pericardial xenograft conduit was selected according to patient anatomy and the targeted diameter, then implanted between the right ventricular outflow tract and the pulmonary artery using continuous 5–0/6–0 polypropylene sutures with attention to alignment and avoidance of kinking. De-identified intraoperative photographs illustrate conduit positioning and final anastomoses **[**Fig. 1, Fig. 2**]**. At the end of each procedure, intraoperative transesophageal echocardiography was performed to confirm adequacy of intracardiac repair, conduit patency, and the absence of significant regurgitation or obstruction.

### 2.4 Echocardiographic assessment

Echocardiography was performed perioperatively, at discharge, and during follow-up. The assessment included peak Doppler gradient across the conduit, regurgitation severity (graded none/trivial/mild/moderate/severe), presence of calcification, conduit dilatation, and leaflet mobility.

### 2.5 Outcomes and definitions

The primary outcome was clean follow-up, pre-specified as: no mortality; no surgical or catheter-based reintervention on the conduit/RVOT; no ≥moderate conduit regurgitation; peak Doppler gradient ≤30 mmHg; and no echogenic signs of calcification on echocardiography.

### 2.6 Complication grading

Postoperative complications were classified using the Clavien–Dindo system.

## 3 Results

Between 2019 and 2024, eight patients received a bovine pericardial xenograft valved conduit. Indications for surgery are summarized in **[**Table 3**]**: four patients with TGA/VSD/PS (Rastelli operation), two with tetralogy of Fallot with absent pulmonary valve, one with VSD–PA with confluent PAs/PDA, and one with truncus arteriosus who underwent a redo procedure for RV–PA graft stenosis 13 years after the primary repair. Previous palliative interventions were common, including six.

**Table 3:** Indications for surgery in the case series.

Indications for surgery	Number of patients
TGA/VSD/PS - Rastelli operation	04
TOF Absent pulmonary valve	02
TA (re-do surgery)	01
VSD–PA with confluent PAs/PDA	01

TGA: Transposition of the great arteries, VSD: Ventricular septal defect, PS: Pulmonary stenosis, TOF: Tetralogy of Fallot, TA: Trancus arteriousus, PABS: Pulmonary artery banding, PDA: Patent ductus arteriosus.

Blalock–Taussig shunts and one prior RV–PA conduit **[**Table 4**]**.

**Table 4:** Previous interventions in the case series.

Intervention	n	Diagnosis at admission
Blalock–Taussig shunt (BT shunt)	6	TGA/VSD/PS - Rastelli operation TOF with absent pulmonary valve
Prior RV–PA conduit	1	Conduit stenosis

Abbreviations: RV–PA, right ventricle to pulmonary artery.

All patients were followed for 6 months to 5 years (mean 2 years), with serial echocardiographic assessments conducted perioperatively, at discharge, and during follow-up. Echocardiography evaluated peak Doppler gradient across the conduit, regurgitation severity (graded none/trivial/mild/moderate/severe), calcification, conduit dilatation, and leaflet mobility. At the latest available follow-up, no patient demonstrated conduit calcification, conduit dilatation, or ≥ moderate regurgitation.

Doppler gradients ranged from 5 to 8 mmHg intraoperatively to 8–12 mmHg at follow-up, and no patient exceeded a peak gradient of 30 mmHg. Leaflet mobility was preserved in all cases.

As shown in **[**Table 5**]**, all eight patients met the definition of clean follow-up, which was pre-specified as: absence of mortality; no surgical or catheter-based reintervention on the conduit/RVOT; no ≥moderate conduit regurgitation; peak.

**Table 5:** Aggregate follow-up outcomes.

Outcome	Value
Follow-up duration	6–60 months (mean 24 months)
Peak Doppler gradient at last follow-up	8–12 mmHg
≥Moderate regurgitation	0/8
Conduit calcification	0/8
Reintervention (surgical/catheter)	0/8
Mortality	0/8

Doppler gradient ≤30 mmHg; and no echogenic signs of calcification.

No postoperative complications were observed; any adverse events would have been classified using the Clavien–Dindo grading system.

### 3.1 Data completeness

Individual patient-level echocardiographic data could not be fully retrieved due to disrupted medical records and loss of contact with some families in the context of ongoing conflict and resource constraints; therefore, outcomes are presented in aggregate.

## 4 Discussion

Reconstructing the RVOT remains essential when RV–PA continuity is disrupted, and the availability of valved conduits has enabled repair of complex congenital lesions that were previously far more difficult to address [3]. In this single-center pediatric case series, RV–PA continuity was restored using a **bovine pericardial xenograft valved conduit (NeoCor)**, which demonstrated encouraging short-term hemodynamic outcomes. All eight patients met the pre-specified definition of clean follow-up, with low perioperative Doppler gradients (5–8 mmHg), stable gradients at follow-up (8–12 mmHg), and no ≥moderate regurgitation or echocardiographic evidence of calcification. These observations support the technical feasibility of a xenograft-based approach in our setting, while acknowledging that durability cannot be inferred from short-term data alone. Despite advances, conduit choice remains complex and context-dependent, influenced not only by age, weight, and anatomy but also by availability, cost, and durability [4,9]. Reviews of RV–PA connections underscore that no single option fulfills all ideal criteria (durability, growth potential, availability) and that conduit exchange remains inevitable over time. Homografts are well established but can be limited by calcification and restricted availability of small sizes, particularly in younger children [[5], [6], [7]]. These constraints—and the impact of size on longevity—are highlighted in long-term homograft series (e.g., Hannover cohort), where degeneration and size-related outcomes are central concerns. Commercial xenografts such as bovine jugular vein (Contegra) offer practical sizing across small diameters and are widely used, yet access and costs vary by region; comparative 20-year data show acceptable survival and reintervention profiles across homografts, Contegra, and decellularized conduits, with smaller sizes and certain diagnoses predicting earlier reintervention [4,9,15]. In parallel, **hand-made valved conduits (e.g., ePTFE)** have been reported with favorable early-to-mid-term outcomes, supporting the acceptability of a hand-constructed strategy in selected contexts when procurement or sizing is constrained [8,16]. From an operative standpoint, successful implantation of a xenograft conduit depends on appropriate conduit sizing and length, careful alignment of the RV–PA pathway, secure anastomoses, and avoidance of kinking or torsion. Intraoperative transesophageal echocardiography is valuable to confirm patency and valve competence immediately after implantation. Pragmatic context matters. Perspectives from low- and middle-income settings emphasize how availability and costs shape congenital cardiac services and timeliness of care [10]. In Syria, conflict-related sanctions and systematic attacks on the health sector have disrupted infrastructure, depleted supplies, and impeded longitudinal follow-up and medical record completeness—factors that explain our reliance on aggregate rather than patient-level reporting in this series [17,18]. This study has limitations: a small retrospective case series without a comparator group; heterogeneous diagnoses and conduit sizes; and variable follow-up durations. Moreover, individual patient-level echocardiographic datasets could not be fully retrieved due to disrupted records and loss of contact in the context of ongoing conflict and resource constraints; thus, outcomes are presented in aggregate. These constraints limit generalizability and preclude firm conclusions about durability or comparative performance versus other conduits [11,15].

In summary, within resource-limited environments, a **bovine pericardial xenograft valved conduit** presents a viable alternative for RV–PA reconstruction when standard conduits are unavailable or unaffordable [4,9,10]. Future efforts should adopt standardized complication reporting, systematic echocardiographic surveillance, and prospective multicenter studies or registry participation to evaluate long-term performance, reintervention rates, calcification, and cost-effectiveness relative to homografts and commercial xenografts [11,15].

## 5 Conclusion

A **pre-fabricated bovine-pericardial valved conduit** (NeoCor patch) is a feasible and accessible option for RV-PA reconstruction, with encouraging short-term hemodynamics in this small single-center series. Given the limited sample size and follow-up duration, durability cannot be determined; systematic echocardiographic surveillance and standardized complication reporting are needed. Prospective multicenter studies—and ideally registry participation—should evaluate long-term performance, reintervention rates, calcification, and cost-effectiveness versus homografts and commercial xenografts, particularly in resource-limited settings.

## Author contribution

**Rahaf Massoud:** Data collection, Manuscript writing, Manuscript editing.

**Sarah Massoud:** Data collection, Manuscript writing.

**Yaman Al-Mkaky:** Data collection, Manuscript writing.

**Yaman Wazir:** Data collection, Manuscript writing.

**Yahya Ibrahim:** Data collection, Manuscript writing.

**Alwaleed Al-Dairy:** Supervision.

## Consent

Written informed consent was obtained from all patients' parents/guardians for publication and any accompanying images. A copy of the written consent is available for review by the Editor-in-Chief of this journal on request.

## Ethical approval

Our institution does not require ethical approval for case reports or case series.

## Guarantor

Rahaf Massoud.

## Sources of funding

None.

## Declaration of competing interest

The authors have no conflict of interest.

## References

[1] Hoffman J.I.E., Kaplan S. (2002). The incidence of congenital heart disease. J. Am. Coll. Cardiol..

[2] Zeng Y.H., Calderone A., Rousseau-Saine N. (2021). Right ventricular outflow tract obstruction in adults: a systematic review and meta-analysis. CJC Open.

[3] Ali Y.A., Roushdy A., Hegab M.A., Mohammed A.M. (2023). Post-right ventricle to pulmonary artery conduit: short- and intermediate-term outcomes: a single-center study. Cardiothorac. Surg..

[4] Salem A.M. (2016). Right ventricle to pulmonary artery connection: evolution and current alternatives. J. Egypt. Soc. Cardio-Thorac. Surg..

[5] Boethig D., Goerler H., Westhoff-Bleck M. (2007). Evaluation of 188 consecutive homografts implanted in pulmonary position after 20 years. Eur. J. Cardiothorac. Surg..

[6] Javadpour H., Veerasingam D., Wood A.E. (2002). Calcification of homograft valves in the pulmonary circulation—is it device or donation related?. Eur. J. Cardiothorac. Surg..

[7] Huyan Y., Chang Y., Song J. (2021). Application of homograft valved conduit in cardiac surgery. Front. Cardiovasc. Med..

[8] Luo K., Zhang Q.L., Zhang X.Y. (2024). Pediatric RVOT reconstruction with ePTFE trileaflet valved conduits: a dual-center Chinese study. Front. Cardiovasc. Med..

[9] Singh S.K., Faridmoayer E., Vitale N. (2025). Valved conduits for right ventricular outflow tract reconstruction: a review of current technologies and future directions. Pediatr. Cardiol..

[10] Sivalingam S. (2024). Outcome of surgery for congenital heart disease – a perspective from Malaysia. Ann. Pediatr. Cardiol..

[11] Mohammadi S., Belli E., Martinovic I. (2005). Surgery for right ventricle to pulmonary artery conduit obstruction: risk factors for further reoperation. Eur. J. Cardiothorac. Surg..

[12] Kerwan A., Al-Jabir A., Agha R. (2025). Revised surgical CAse REport (SCARE) guideline: an update for the age of artificial intelligence [internet]. Prem J. Sci..

[13] Mathew G., Agha R., Kerwan A. (2025). Revised preferred reporting of case series in surgery (PROCESS) guideline: an update for the age of artificial intelligence [internet]. Prem J. Sci..

[15] Sabateen F., Soják V., Nagi A.S., Valentík P., Šagát M., Nosál’ M. (2023). 20-year follow-up and comparison of valved conduits used for right ventricular outflow tract reconstruction: single-centre, propensity score match analysis. Interact. Cardiovasc. Thorac. Surg..

[16] Çiçek M., Özdemir F., Yurdakök O. (2025). Comparison of bicuspid and tricuspid handmade polytetrafluoroethylene valved conduits: early and mid-term results. J. Clin. Med..

[17] Sen K., Al-Faisal W., AlSaleh Y. (2013). Syria: effects of conflict and sanctions on public health. J. Public Health.

[18] Fouad F.M., Sparrow A., Tarakji A. (2017). Health workers and the weaponisation of health care in Syria: a preliminary inquiry for The Lancet–American University of Beirut commission on Syria. Lancet.

